# Supplementary material for: Biochemical and Structural Characterization of Chi-Class Glutathione Transferases: A Snapshot on the Glutathione Transferase Encoded by sll0067 Gene in the Cyanobacterium Synechocystis sp. Strain PCC 6803
Source: Biomolecules. 2022 Oct 13;12(10):1466. doi: 10.3390/biom12101466 (PMC9599700; doi:10.3390/biom12101466)
Supplement: Supplementary file 1 [file biomolecules-12-01466-s001.zip › biomolecules-1876890- Supplementary.pdf]

Supplementary Materials

Biochemical and Structural Characterization of Chi-Class Glutathione Transferases: A Snapshot on the Glutathione Transferase Encoded by *sll0067* Gene in the Cyanobacterium *Synechocystis* sp. Strain PCC 6803

E. Mocchetti & L. Morette *et al.*

Table of contents

Supplementary methods on the structure analysis based on electron density distribution. 3

Table S1. List of the PCR and mutagenic primers used in this study. 4

Table S2. List of the 222 studied proteomes. 5

Table S3. List of the 53 ribosomal protein families present in bacteria according to riboDB database (https://umr5558-bibiserv.univ-lyon1.fr/riboDB/ribodb.cgi). 6

Table S4. Strong intersubunit contacts in SynGSTC1. 7

Table S5. Comparison of SynGSTC1 with its structural homologs. 8

Table S6. Permanent, polarization and total electrostatic interaction energies in the active sites of SynGSTC1, of Epsilon 2 GST from *Anopheles Gambiae* (AgGSTE2) and of Alpha 1 GST from chicken (GgGSTA1). 9

Table S7. Kinetic parameters of SynGSTC1 toward model substrates. 11

Table S8. List of the 147 full-length protein sequences displaying a SRAS motif (or a related motif) identified in the 222 proteomes of Cyanobacteria/Melainabacteria group. 12

Table S9. Invariant amino acid residues in the GST Chi Class. 13

Table S10. Multiple sequence alignment of the 147 GSTCs (displaying a SRAS motif or a related motif) identified in the 222 proteomes of Cyanobacteria/Melainabacteria group. 14

Figure S1. Stereoviews of the 2mFo-DFc map of the SynGSTC1 inter-domain linker. 15

Figure S2. Φ and Ψ torsion angles for the inter-domain linker residues in SynGSTC1 during the simulation. 16

Figure S3. Structure-based phylogenetic tree of SynGSTC1 with structural homologs. 18

Figure S4. Stereoview of the comparison of the SRAS motif in SynGSTC1 and in GST SMc00097 from *Sinorhizobium meliloti 2011*. 19

Figure S5. N-Cα-Cβ-Sγ torsion angle of glutathione during molecular dynamics simulation of SynGSTC1. 20

Figure S6. Interatomic distances (Å) between γ-oxygen atom of Ser10 and selected atoms during molecular dynamics simulation of SynGSTC1. 21

Figure S7. Optimal reaction pH of SynGSTC1 and variants S10T, S10A, S10C and R11A. 22

Figure S8. Structural comparison of the active site of SynGSTC1 WT with S10T and R11A variants. .. 23

Figure S9. Phylogeny of the 870 GST sequences identified in the 222 studied proteomes of the Cyanobacteria / Melainabacteria group and the 11 GSTC-related sequences identified in non-cyanobacterial bacteria (881 sequences, 104 amino acid positions)... 24

Figure S10. Phylogeny of the 147 cyanobacterial GST1 sequences harboring the SRAS motif or related motifs... 26

Figure S11. Phylogeny of the 222 proteomes of Cyanobacteria / Melainabacteria group considered in this study (high quality version of the tree provided in Figure 5).. 28

Figure S12. Stereoview of the invariant amino acid residues in the GST Chi Class and WebLogos of aligned GSTCs from cyanobacteria.. 30

References. 31

Supplementary methods on the structure analysis based on electron density distribution.

To calculate the electrostatic interaction energies between residues of SynGSTC1 active site and glutathione, the charge density of the complex based on the Hansen and Coppens multipolar model [1] has been determined. This model sums atomic contributions, divided in point charge treatment for nuclei and continuous distribution for electron density which is described with three parametrized terms:

$$\rho_{\text{atom}}(\boldsymbol{r}) = \rho_{\text{core}}(r) + P_{\text{val}}\kappa^3\rho_{\text{val}}(\kappa, r) + \sum_{l=0}^{l_{\max}} R_l(\kappa', r)\kappa'^3 \sum_{m=-l}^{l} P_{lm} d_{lm}(\theta, \varphi).$$

The first and second terms correspond to the core and valence spherical contributions respectively. The last one is a multipolar term enabling to reproduce the asphericity of the valence electron density. Electron density parameters in this model are the atomic valence populations (P_{val}), representing atomic charges and the multipole populations P_{lm}. The parameters κ and κ' allow to modulate the contraction or the expansion of the spherical and deformation valence shell, respectively. These density parameters for the SynGSTC1-GSH complex have been transferred from the ELMAM2 database [2] which provides parameters averaged over experimental peptide electron densities from high resolution X-Ray scattering data. To perform this transfer, the transfer tool of the *MoProViewer* software [3] has been used. For that purpose, hydrogen atoms have been added to the structure using the MolProbity server [4] and the protonation state of the glutathione has been adjusted manually using *PyMol* [5]. The MoProViewer database transfer tool enables an automatic parameter transfer on the structure with appropriate formal charge assignment (+1e for arginine and lysine, -1e for aspartate and glutamate, 0 for others). The His38 and His61 of SynGSTC1 have been protonated on Nε atom and the formal charge of glutathione has been set to -1e.

In addition to the electron density parameters, anisotropic atomic polarizabilities have also been transferred to the complex from the ELMAM2 database. To account for polarization effects in the transferred electron density due to the environment, the procedure described in Leduc *et al.* [6] has been followed thanks to the *MoProViewer* Polarizer tool. This method consists in mutual intermolecular polarization, here between the glutathione ligand and the active site residues. Atomic dipoles are being induced in each group due to electric fields emanating from the other, in an iterative way. Convergence was considered reached when all induced atomic dipoles in a given polarization cycle are smaller than 10^{-3} e.Å.

Thus, the electrostatic interaction permanent energy E_{perm}^{elec} and the total electrostatic interaction energy E_{tot}^{elec}, which also include the polarization contribution E_{pol}^{elec} (hence $E_{tot}^{elec} = E_{perm}^{elec} + E_{pol}^{elec}$), have been computed between the glutathione and the SynGSTC1 active site residues. For that purpose, the fast and analytical electrostatic energy calculation tool *Charger* [7] has been used. This tool is based on the aEP/pMM (analytical exact potential / pseudo-multipolar moments) method and is implemented in *MoProViewer*. The calculations have been performed for the eleven residues around the glutathione which have atoms at a distance less than 3.5 Å away from the glutathione. This includes eight residues from one chain (Arg11, Leu33, His38, Lys51, Val52, Glu64, Ser65 and Asn97) and three from the other (Ser98, Thr99 and Arg116). The results for $E_{\text{perm}}^{\text{elec}}$, $E_{\text{pol}}^{\text{elec}}$ and $E_{\text{tot}}^{\text{elec}}$ presented in this study are averaged over the homodimer.

Table S1. List of the PCR and mutagenic primers used in this study.

SynGSTC1-*Nde*I For
5′-ggggggCATATGatcaaactatacggtgc

SyngGSTC1-*Xho*I Rev
5′-ggggggCTCGAGtcagcgggcaccgatggaag

SynGSTC1-S10T For
5'-atacggtgccccccaaactcgagcctccatc-3'

SynGSTC1-S10T Rev
5'-gatggaggctcgagtttggggggcaccgtat-3'

SynGSTC1-S10A For
5'-atacggtgccccccaagctcgagcctccatc-3'

SynGSTC1-S10A Rev
5'-gatggaggctcgagcttggggggcaccgtat-3'

SynGSTC1-S10C For
5'-atacggtgccccccaatgtcgagcctccatc-3'

SynGSTC1-S10C Rev
5'-gatggaggctcgacattggggggcaccgtat-3'

SynGSTC1-R11A For
5'-atacggtgccccccaaagtgcagcctccatc-3'

SynGSTC1-R11A Rev
5'-gatggaggctgcactttggggggcaccgtat-3'

Table S2. List of the 222 studied proteomes.
This table is in a separate Microsoft Excel file.

Table S3. List of the 53 ribosomal protein families present in bacteria according to riboDB database (https://umr5558-bibiserv.univ-lyon1.fr/riboDB/ribodb.cgi).
This table is in a separate Microsoft Excel file.

Table S4. Strong intersubunit contacts in SynGSTC1.

Two-fold axis interface		Distance (Å) A-B	Distance (Å) B-A
P48-O	S120-OG	2.85	2.77
K51-NZ	E113-OE2	2.76	2.85
W63-N	Q91-OE1	2.99	3.00
E64-OE1	S98-OG	3.28	3.26
E64-OE2	T99-OG	2.59	2.67
Y71-OH	Q84-NE2	3.11	3.17
E74-OE1	R86-NH2	2.93	3.02
E74-OE2	R86-NE	2.67	2.63

Table S5. Comparison of SynGSTC1 with its structural homologs.

The selected homologs are GST nu from Escherichia coli K-12 (PDB entry 5HFK), Ure2p5 from *Phanerodontia chrysosporium* (PDB entry 4F0C), GST beta from *Methylococcus capsulatus* str. Bath (PDB entry 3UAP), GST from *Sinorhizobium meliloti* 2011 (PDB entry 4NHW), GST delta 2 from *Drosophila melanogaster* (PDB entry 5F0G) and GST phi 8 from *Populus trichocarpa* (PDB entry 5F07). The sequence motifs are in the same position as the CXXC catalytic motif of thioredoxins, i.e. at the N-terminus of α1 helix. The subunit of SynGSTC1 was superimposed with that of its homologs. The N- and C-terminal domains were also compared. The calculated rmsds (root mean square deviations) (Å) and the numbers of superimposed Cα atoms are given in the last three lines.

		**Nu class**	**Ure2p class**	**Beta class**	**Unclassified**	**Delta class**	**Phi class**
	PDB	5HFK	4F0C	3UAP	4NHW	5F0G	5F07
	Motif	^{9}TPNG^{12}	^{15}GPNG^{18}	^{9}ACSL^{12}	^{10}SRAS^{13}	^{9}GGGC^{12}	^{11}AVCP^{14}
**Rmsd (Å) / Nb of Cα)**	Overall	1.37/128	1.39/121	2.35/151	1.02/119	1.61/108	1.32/119
	N-ter domain	0.59/66	0.50/55	0.88(57)	0.78(61)	0.91(45)	0.72(65)
	C-ter domain	1.68/68	1.43/64	2.36(87)	0.88(60)	2.58(79)	1.90(64)

Table S6. Permanent, polarization and total electrostatic interaction energies in the active sites of SynGSTC1, of Epsilon 2 GST from *Anopheles Gambiae* (AgGSTE2) and of Alpha 1 GST from chicken (GgGSTA1).

The permanent $E_{\mathrm{perm}}^{\mathrm{elec}}$, polarization $E_{\mathrm{pol}}^{\mathrm{elec}}$ and total $E_{\mathrm{tot}}^{\mathrm{elec}}$ electrostatic interaction energies between glutathione and active-site residues are presented in kcal/mol. $E_{\mathrm{perm}}^{\mathrm{elec}}$ was computed using the electron density model transferred on the glutathione and the protein atoms, whereas E_{tot}^{elec} was obtained after the electron density polarization procedure. Finally, $E_{\mathrm{pol}}^{\mathrm{elec}}$ was computed using $E_{\mathrm{pol}}^{\mathrm{elec}} = E_{tot}^{elec} - E_{\mathrm{perm}}^{\mathrm{elec}}$. $E_{\mathrm{pol}}^{\mathrm{elec}}$ represents the polarization contribution to the total electrostatic interaction energy.

SynGSTC1

**Residue**	$\boldsymbol{E_{\mathrm{tot}}^{\mathrm{elec}}}$	$\boldsymbol{\sigma(E_{\mathrm{tot}}^{\mathrm{elec}})}$	$\boldsymbol{E_{\mathrm{perm}}^{\mathrm{elec}}}$	$\boldsymbol{\sigma(E_{\mathrm{perm}}^{\mathrm{elec}})}$	$\boldsymbol{E_{\mathrm{pol}}^{\mathrm{elec}}}$	$\boldsymbol{\sigma(E_{\mathrm{pol}}^{\mathrm{elec}})}$
Arg11	-56.6	3.6	-47.3	2.7	-9.3	0.9
Ser65	-54.3	2.4	-39.3	1.6	-15.0	0.8
Ser98*	-4.5	0.1	-2.2	0.1	-2.3	0.0
Thr99*	-8.7	0.4	-5.3	0.2	-3.4	0.1
Glu64	-6.0	0.6	13.6	0.6	-19.7	0.1
Asn97	2.2	0.1	3.5	0.2	-1.3	0.1
Val52	-27.4	1.3	-18.3	0.7	-9.1	0.7
Ser10	1.3	0.3	1.3	0.3	0.1	0.3
Leu33	-3.5	0.7	-2.8	0.3	-0.7	0.4
His38	-21.5	0.8	-14.7	0.3	-6.7	0.5
Arg116*	-66.7	4.2	-58.6	2.9	-8.1	1.3
Lys51	-134.9	2.7	-105.2	1.0	-29.7	1.7

The interaction energies have been averaged over the two conformations of glutathione (A and B) observed in the crystal structure and over the two monomers. For each energy value, the associated standard deviation σ is given in the adjacent column. The residues marked with an asterisk (*) in the table are from the other monomer than the glutathione. SynGSTC1 is a SerGST because it contains a serine residue (S10) at the N-terminal end of α1 helix. Surprisingly S10 has no significant electrostatic influence on glutathione.

GgGSTA1			
**Residues**	$E_{\text{tot}}^{\text{elec}}$	$E_{\text{perm}}^{\text{elec}}$	$E_{\text{pol}}^{\text{elec}}$
Lys15	-66.3	-53.1	-13.3
Thr68	-57.3	-31.9	-25.4
Gln67	-15.1	-9.0	-6.1
Asp101*	-13.9	4.9	-18.8
Pro56	-5.5	-0.9	-4.6
Val55	-13.9	-10.1	-3.8
Tyr9	-12.1	-9.0	-3.1
Phe10	-2.5	-2.3	-0.1
Gln54	-53.4	-32.0	-21.4
Tyr41	-1.0	0.5	-1.6
Leu220	-1.9	-1.9	0.0
Met222	-3.7	-1.0	-2.7
Tyr223	-9.6	-7.7	-1.8
Arg131*	-114.8	-89.6	-25.3

No average has been made to compute the energies for Alpha 1 GST from chicken (GgGSTA1, pdb entry 1VF1) since the asymmetric unit contains a monomer. The residues marked with an asterisk (*) in the table are from a symmetry related monomer than the glutathione. GgGSTA1 is a TyrGST because it contains a tyrosine residue (Y9) at the C-terminal end of $\beta 1$ helix. Y9 has an electrostatic influence on the thiol group of glutathione as expected.

AgGSTE2			
**Residues**	$E_{\text{tot}}^{\text{elec}}$	$E_{\text{perm}}^{\text{elec}}$	$E_{\text{pol}}^{\text{elec}}$
Ser68	-26.1	-18.0	-8.1
Glu67	5.5	20.3	-14.8
His69	-2.3	-3.4	1.2
Pro14	-9.9	-7.1	-2.8
His101*	-0.2	-0.5	0.4
Pro56	-3.3	-0.2	-3.1
Phe108	-8.8	-4.4	-4.5
Ile55	-14.1	-7.9	-6.2
Ser12	-7.3	-5.9	-1.4
Pro13	0.9	1.7	-0.8
Leu36	-4.2	-3.5	-0.7
Thr54	-22.6	-14.9	-7.6
His41	-12.5	-9.3	-3.2
His53	-11.3	-4.1	-7.2
Arg112	-124.9	-91.4	-33.5

The energies have been averaged over the dimer for Epsilon 2 GST from *Anopheles Gambiae* (AgGSTE2, pdb entry 2IMI). The residues marked with an asterisk (*) in the table are from the other monomer than the glutathione. AgGSTE2 is a SerGST because it contains a serine residue (S12) at the N-terminal end of $\alpha 1$ helix. S12 has an electrostatic influence on the thiol group glutathione thiol group.

Table S7. Kinetic parameters of SynGSTC1 toward model substrates.

	**CDNB**	**BITC**	**PITC**	**PNP-butyrate**	**CuOOH**
$\boldsymbol{k_{cat}}$ **(s^{-1})**	0.64 ± 0.16	46.94 ± 1.60	21.00 ± 0.54	0.48 ± 0.2	0.91 ± 0.06
$\boldsymbol{K_m}$ **(µM)**	3786.0 ± 772.2	82.0 ± 9.9	31.4 ± 3.4	162.5 ± 18.3	1508.0 ± 191.0
$\boldsymbol{k_{cat}/K_m}$ **($M^{-1}.s^{-1}$)**	112.5 ± 14.2	$5.7 \times 10^5 \pm 0.2 \times 10^5$	$6.7 \times 10^5 \pm 0.2 \times 10^5$	49.0 ± 1.7	604.6 ± 37.7

The apparent K_m values of SynGSTC1 were determined by varying substrate concentrations at a fixed saturating GSH concentration. The apparent K_m and k_{cat} values were calculated with *GraphPad Prism 8* software using the Michaelis–Menten equation as non-linear regression model. Results are means ± S.D. (n = 3).

Table S8. List of the 147 full-length protein sequences displaying a SRAS motif (or a related motif) identified in the 222 proteomes of Cyanobacteria/Melainabacteria group.
This table is in a separate Microsoft Excel file.

Table S9. Invariant amino acid residues in the GST Chi Class.
Numbering of residues is according to sequence of SynGSTC1.

Residue	Environment, possible function, and effects of mutations	References
R11	In α1-β1 loop at active site; stabilize GSH; replacement with A in GSTH1-1 Eta from *Agrobacterium tumefaciens* has detrimental effect on activity.	[8]
E21	In α1; hydrogen bonded to the side chains of W17 (N-ter domain) and R174 (C-ter domain); well conserved in the Phi and Ure2p classes	[9-10]
N47	In β2-α2 loop; its lateral chain is hydrogen bonded to main-chain atoms to stabilize the β2-α2 loop; well conserved in the Ure2p and Nu classes.	[9]
P53	Precedes β3 at active site; in a cis-configuration and essential for the stability of the protein; replacement with G in hGSTA1-1 dramatically reduced the catalytic activity.	[11]
E64	Precedes α3 at active site; salt bridged to the terminal NH3 group of GSH ; well conserved in Ser-GSTs	[9]
^{65}SG^{66} & L69	In α3 below the G-site; part of the SNAIL/TRAIL motif well preserved in GSTs; core residues of the N-ter domain; S65 is hydrogen bonded to the carboxylate group of the GSH γ-glutamyl moiety via its main chain and its side chain.	[11]
Q91	In α4 at the dimer interface; hydrogen bonded to the main chain of W63 of the neighboring subunit; well preserved in the tau class.	[10]
W92	In α4 at the dimer interface; in a hydrophobic pocket at the dimer interface; well preserved in the tau and phi classes.	[10]
N97	In α4 at the active site; located near the γ-glutamyl moiety of GSH and close to the guanidinium group of the SRAS motif.	
D140	In α6; quasi invariant residue that belongs to a N-capping box motif at the beginning of α6 in the C-ter domain of GSTs.	[12-13]
L147	In α6; member of hydrophobic core between α5, α6 and α7; well conserved in Alpha, Mu and Pi classes.	[14]

Table S10. Multiple sequence alignment of the 147 GSTCs (displaying a SRAS motif or a related motif) identified in the 222 proteomes of Cyanobacteria/Melainabacteria group.
This table is in a separate text file (fasta format).

Figure S1. Stereoviews of the 2mFo-DFc map of the SynGSTC1 inter-domain linker.

The figure shows the final 2mFo-DFc map in the region of the SynGSTC1 inter-domain linker. The latter was found disordered in two major conformations. The map was calculated using *BUSTER* [15] and the figure was generated by *Coot* [16]. The map was contoured at 1.0 σ level where σ is the standard deviation of the map.

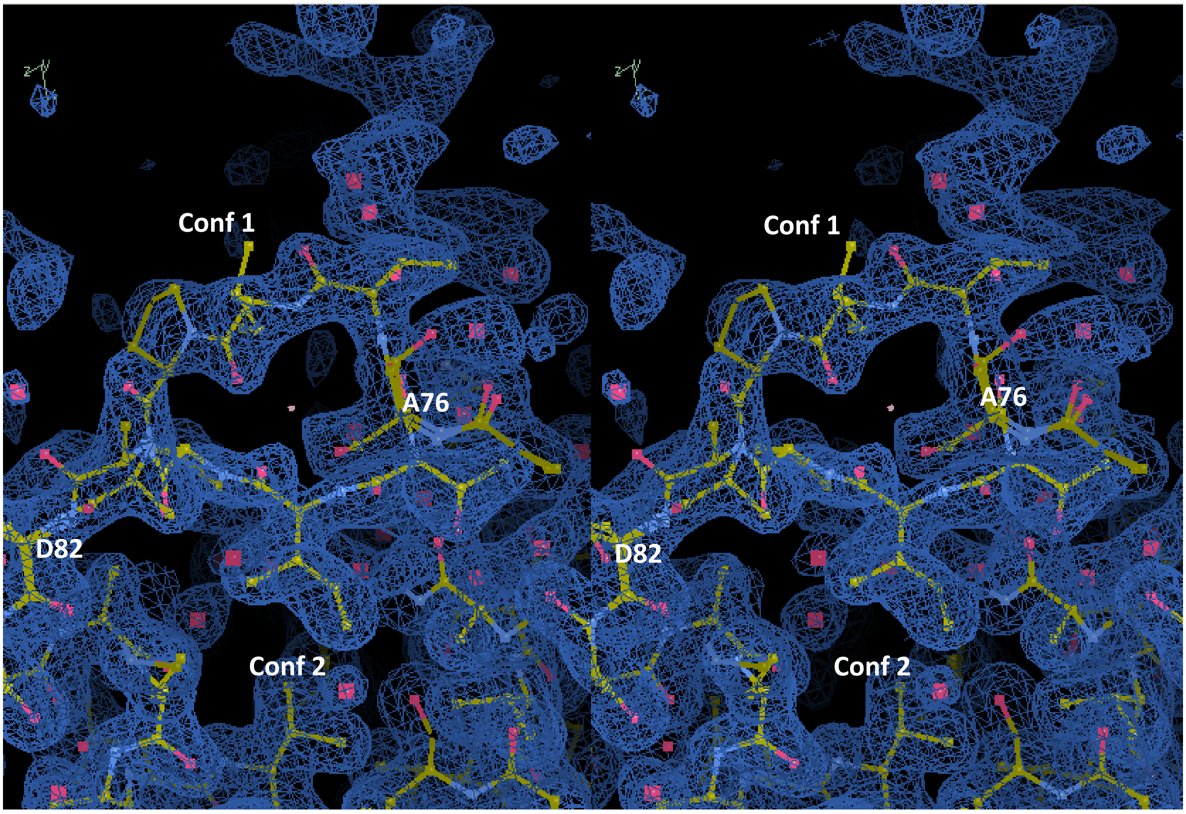

Figure S2. Φ and Ψ torsion angles for the inter-domain linker residues in SynGSTC1 during the simulation.

Measure of the torsions Φ (C(i-1)-N(i)-Cα(i)-C(i)) and Ψ (N(i)-Cα(i)-C(i)-N(i+1)) are represented by blue and green dots respectively along the molecular dynamics trajectory for the residues 76 to 82 in the two monomers of SynGSTC1. The lines correspond to the Φ (dark blue) and Ψ (dark green) values measured in the crystal structure for the two visible conformations of the linker. The vertical axis displays the angle value in degrees (°) and the horizontal axis corresponds to the simulation time in nanoseconds (ns). The dynamics shows leaps, especially distinct for Ala81 and Asp82, suggesting that the simulation reproduce the two conformations observed in the crystal structure.

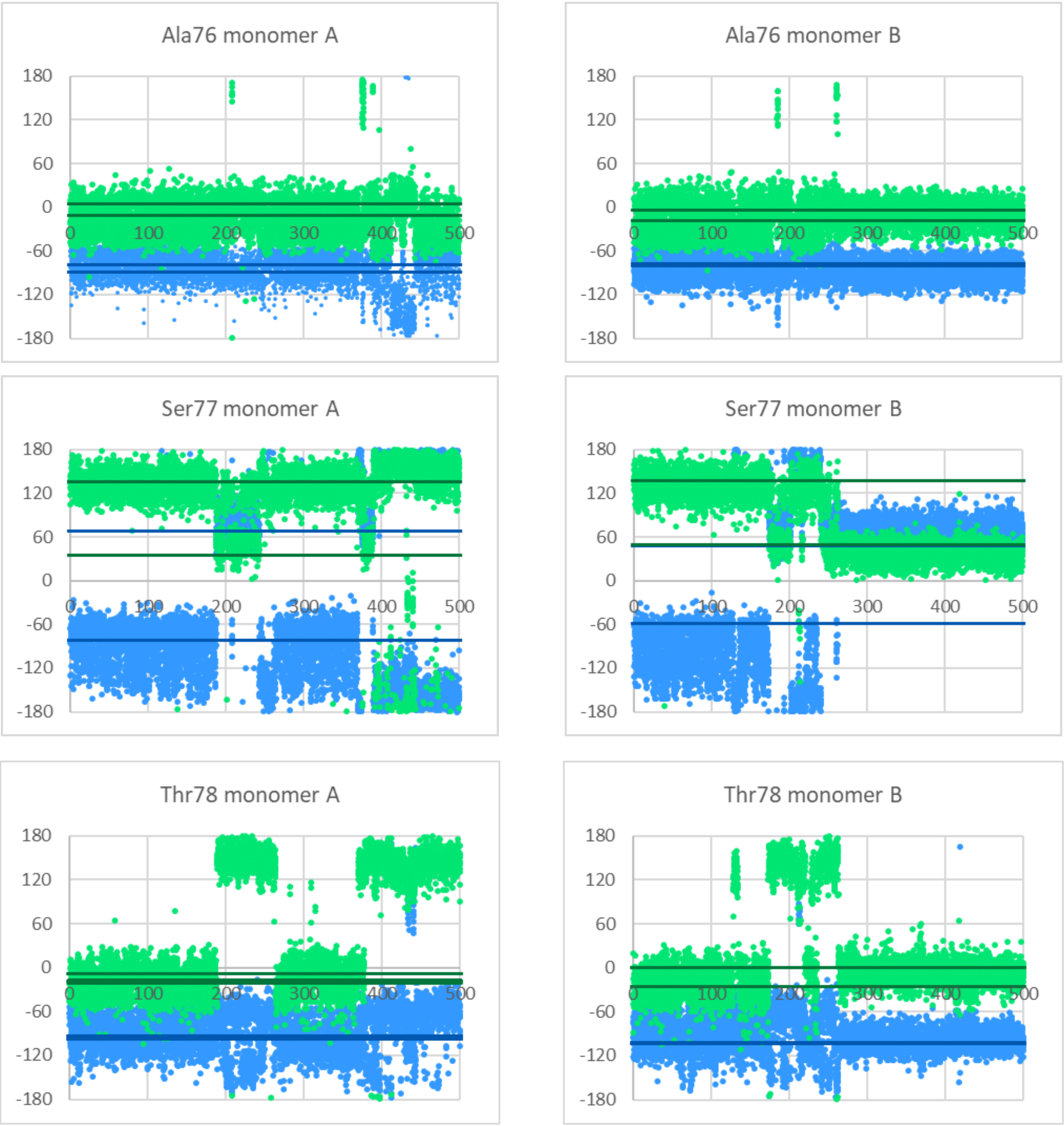

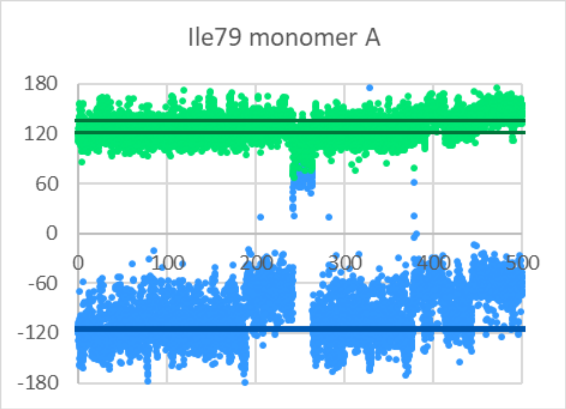
Ile79 monomer A
180
120
60
0
-60
-120
-180
0
100
200
300
400
500

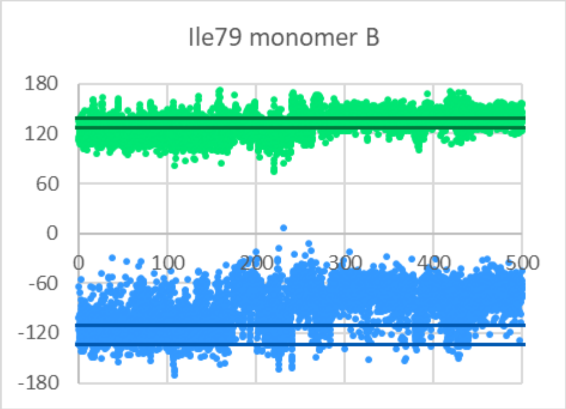
Ile79 monomer B
180
120
60
0
-60
-120
-180
0
100
200
300
400
500

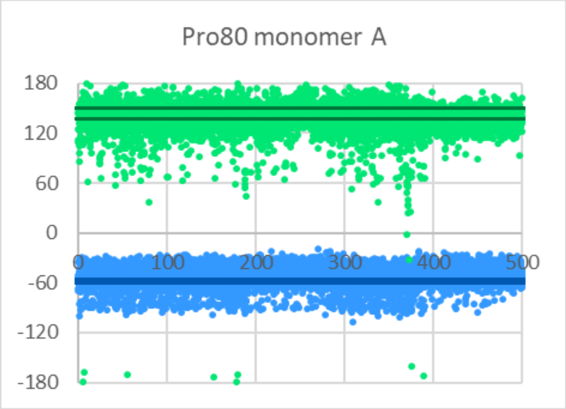
Pro80 monomer A
180
120
60
0
-60
-120
-180
0
100
200
300
400
500

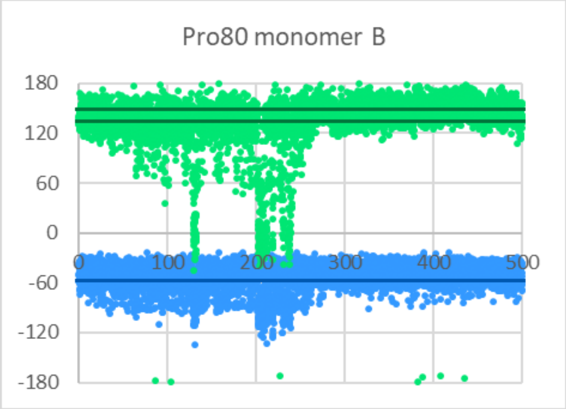
Pro80 monomer B
180
120
60
0
-60
-120
-180
0
100
200
300
400
500

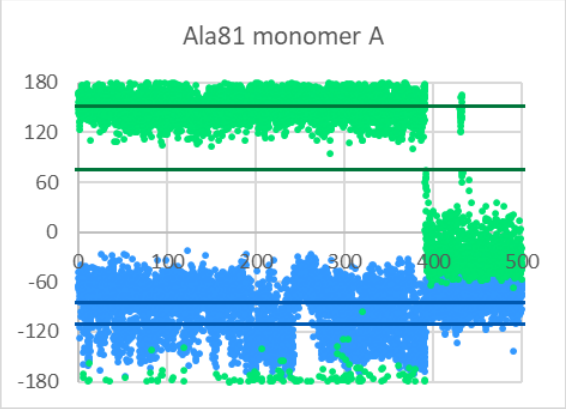
Ala81 monomer A
180
120
60
0
-60
-120
-180
0
100
200
300
400
500

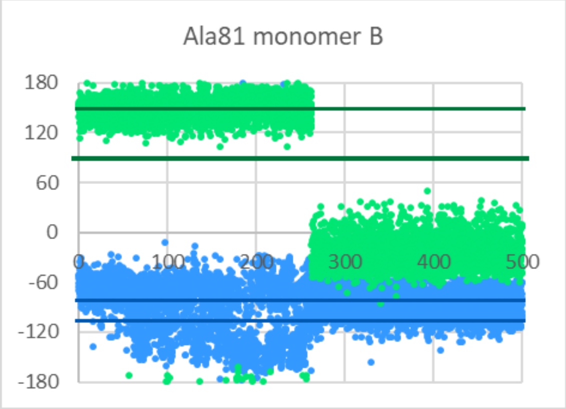
Ala81 monomer B
180
120
60
0
-60
-120
-180
0
100
200
300
400
500

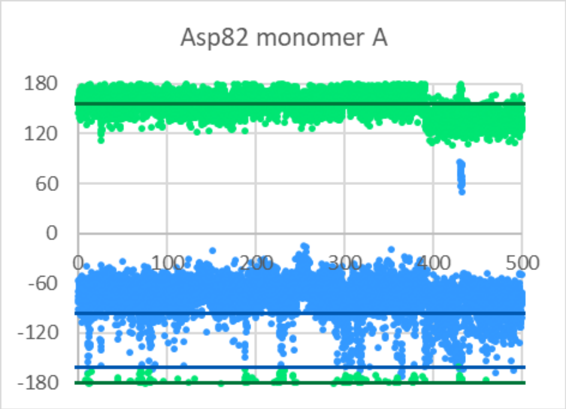
Asp82 monomer A
180
120
60
0
-60
-120
-180
0
100
200
300
400
500

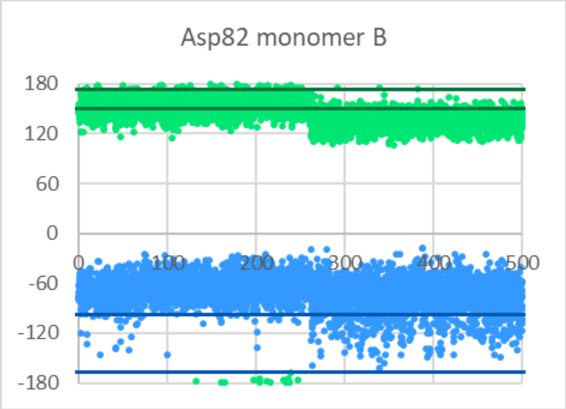
Asp82 monomer B
180
120
60
0
-60
-120
-180
0
100
200
300
400
500

Figure S3. Structure-based phylogenetic tree of SynGSTC1 with structural homologs.

The structure-based phylogenetic tree was generated using the mTM-align server (https://yanglab.nankai.edu.cn/mTM-align/) based on pairwise template modeling scores. Crystal structures and sequences can be found at the Protein Data Bank (http://www.rcsb.org) : SynGSTC1, this study, PDB entry 8AI8; SmGST, GST from *Sinorhizobium meliloti* 2011, PDB entry 4NHW; DmGSTD2, GST delta 2 from *Drosophila melanogaster*, PDB entry 5F0G; McGSTB, GST beta from *Methylococcus capsulatus* str. Bath, PDB entry 3UAP; PtGSTF8, GST phi 8 from *Populus trichocarpa*, PDB entry 5T07; EcYfcG , GST nu from *Escherichia coli* K-12, PDB entry 5HFK; PcUre2p5, Ure2p5 from *Phanerodontia chrysosporium*, PDB entry 4F0C.

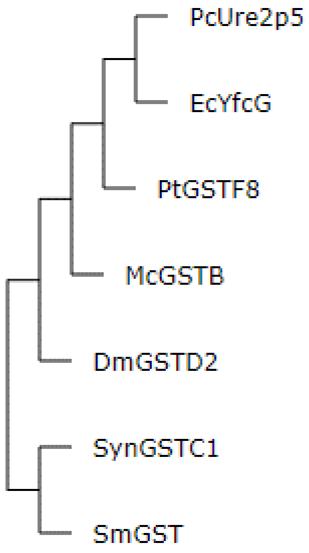

Figure S4. Stereoview of the comparison of the SRAS motif in SynGSTC1 and in GST SMc00097 from *Sinorhizobium meliloti 2011*.

The figure shows that the SRAS motif has the same conformation in SynGSTC1 (green) and in GST SMc00097 from *Sinorhizobium meliloti 2011* (PDB entry 4nhw) (magenta). In both cases the side chain of the first serine residue does not point towards the thiol group of glutathione but towards the interior of the protein.

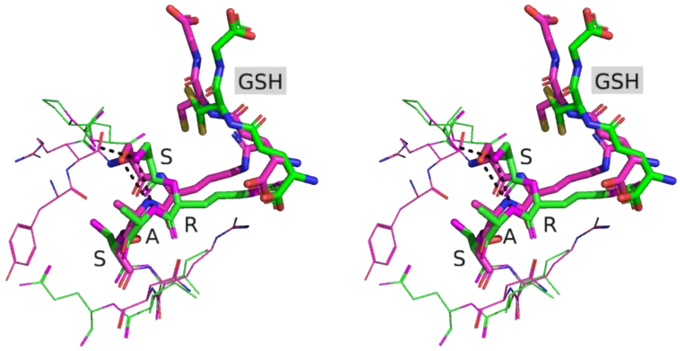

Figure S5. N-Cα-Cβ-Sγ torsion angle of glutathione during molecular dynamics simulation of SynGSTC1. The thiol group of the glutathione indiscriminately adopts the conformations of the three most common rotamers of cysteine. The high frequency of fluctuations between these rotamers suggests that the thiol group is not stabilized by the enzyme. This feature is represented here in the monomer A of SynGSTC1 but it can be observed as well as in the monomer B. The molecular dynamics simulation time is given in nanoseconds (ns) and the N-Cα-Cβ-Sγ torsion angle of in degree (°).

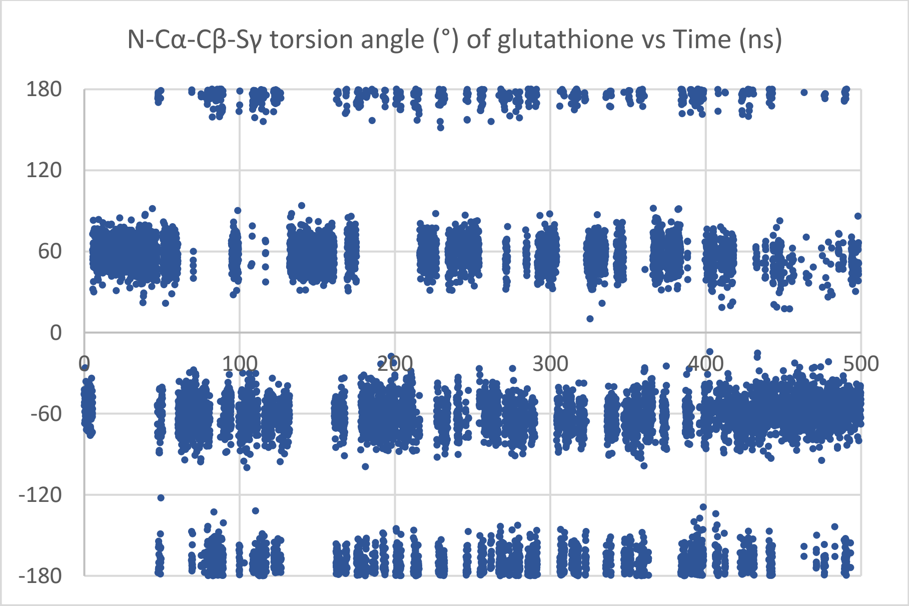

Figure S6. Interatomic distances (Å) between γ-oxygen atom of Ser10 and selected atoms during molecular dynamics simulation of SynGSTC1.

The selected atoms are the sulfur atom Sγ of glutathione (green line), the oxygen atom O and the nitrogen N of Ala7 (blue and red lines, respectively) and the nitrogen atom N of Ala12 (purple line). These measures show that the hydroxyl group of Ser10 shares a strong hydrogen bond with the carbonyl group of Ala7 and weak hydrogen bonds with the amine groups of Ala7 and Ala12. These interactions appear stable during most of the trajectory. In this case the side chain of Ser10 oriented towards the core of the protein. The lateral chain of Ser10 has also the possibility to be oriented toward the solvent. In this rotamer, Ser10 is less distant from the thiol group of glutathione but the distance is still too long (more than 4 Å) for a strong intermolecular interaction. The large fluctuations in the Ser10Oγ-GSHSγ distance suggest that the thiol group does point toward the Ser10 hydroxyl group. The molecular dynamics simulation time is given in nanoseconds (ns) and the distances in angstroms (Å).

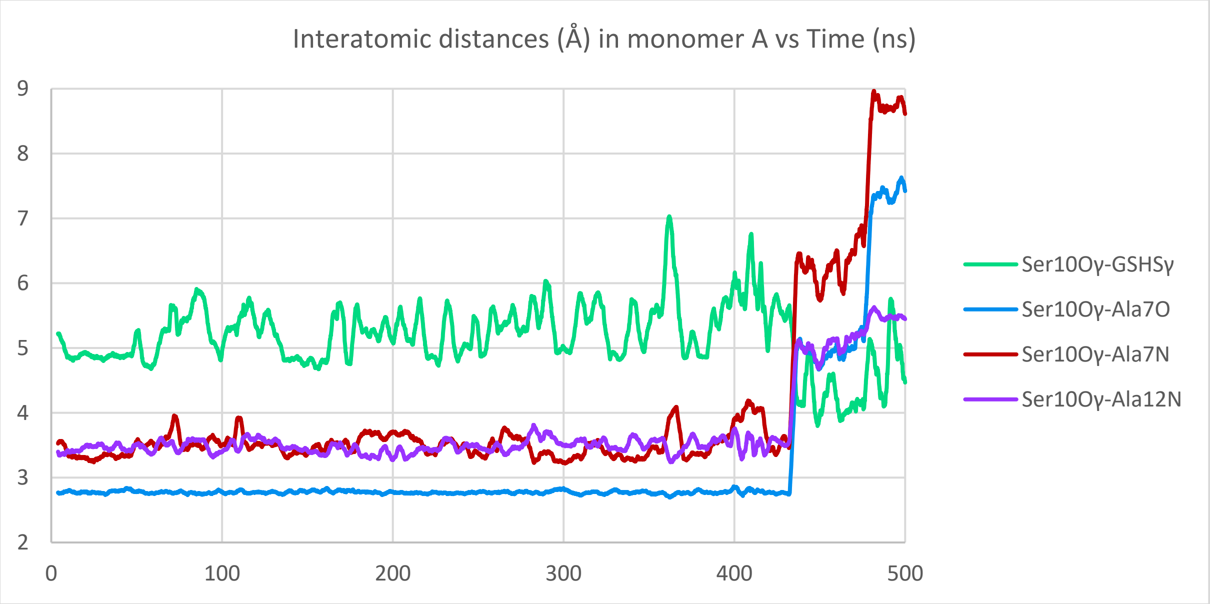

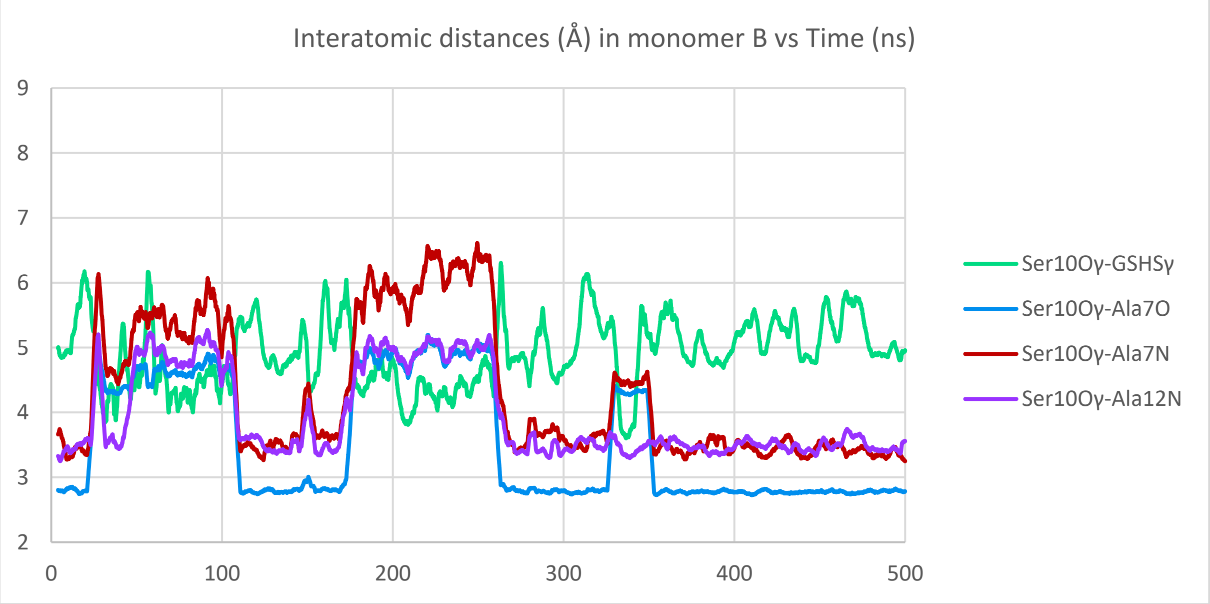

Figure S7. Optimal reaction pH of SynGSTC1 and variants S10T, S10A, S10C and R11A.

The optimum reaction pH of SynGSTC1 and variants S10T, S10A, S10C and R11A was determined at 25°C by GSH-conjugation assays using 2-phenetyl-isothiocyanate (PITC) as substrate. The reactions were performed in 500 µl of 100 mM sodium citrate, phosphate, or borate buffers (pH ranging from 4.0 to 11.0) and a fixed concentration of PITC and GSH of 1 mM. Recombinant proteins used at a concentration of 1 µM were added after 2 min of incubation and the variation of absorbance monitored at 274 nm using a Cary 50 spectrophotometer. The activity recorded without enzyme was subtracted and three independent experiments were performed at each pH.

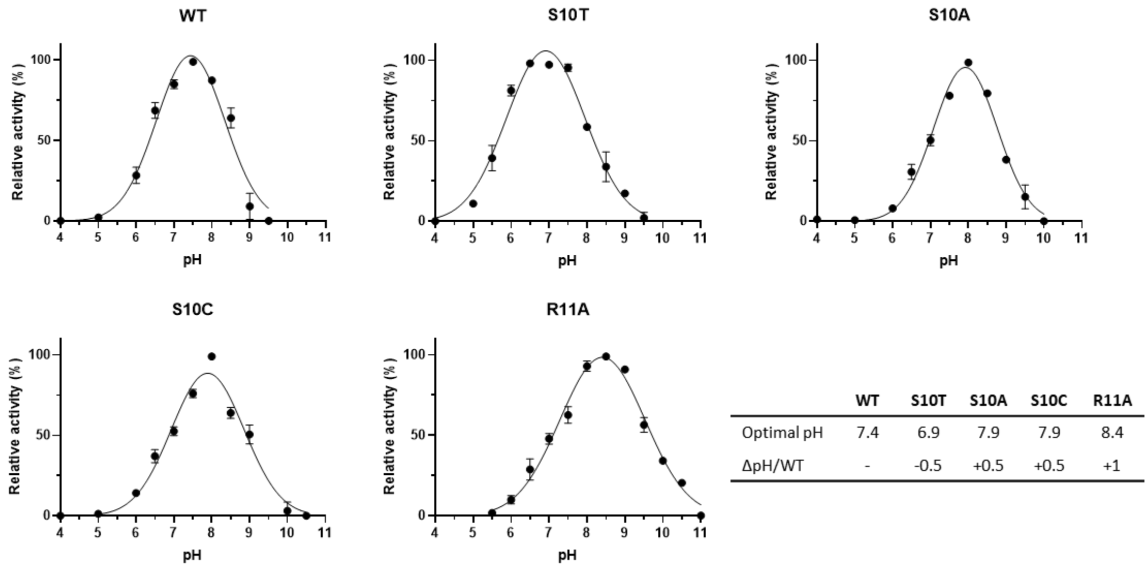

	WT	S10T	S10A	S10C	R11A
Optimal pH	7.4	6.9	7.9	7.9	8.4
ΔpH/WT	-	-0.5	+0.5	+0.5	+1

Figure S8. Structural comparison of the active site of SynGSTC1 WT with S10T and R11A variants. The figures show that the S10T and R11A mutations do not alter the structure of the active site of SynGSTC1.

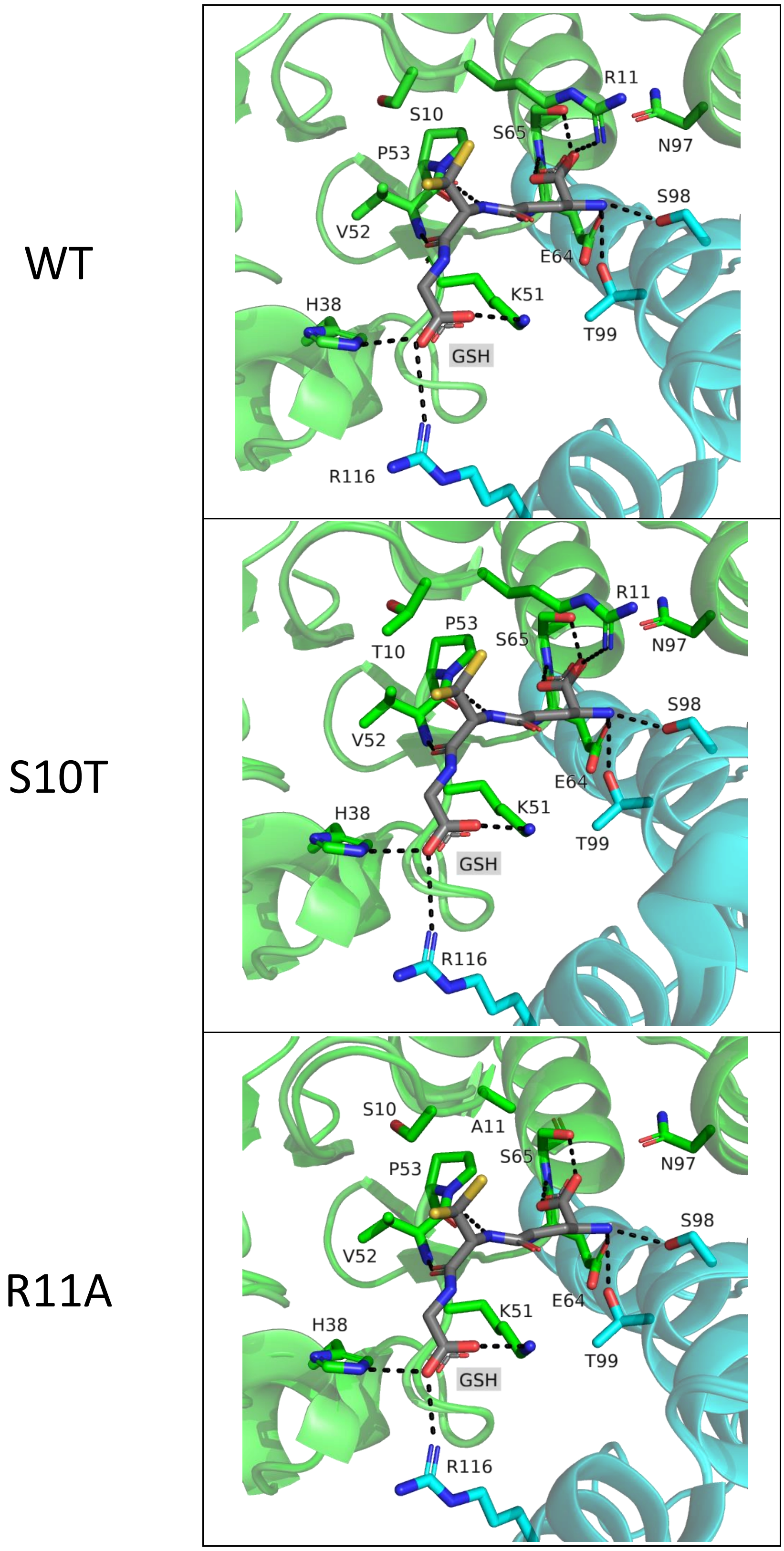

Figure S9. Phylogeny of the 870 GST sequences identified in the 222 studied proteomes of the Cyanobacteria / Melainabacteria group and the 11 GSTC-related sequences identified in non-cyanobacterial bacteria (881 sequences, 104 amino acid positions).

The tree was inferred with *FastTree* [17] using the LG model. The scale bar indicates the average number of substitutions per site. For clarity, only the part of the tree encompassing GSTC sequences is detailed, while other sequences have been collapsed. The 147 cyanobacterial GSTC sequences are shown in pink, while the 11 GSTC-related sequences found in non-cyanobacterial bacteria are in blue. Note that these non-cyanobacterial bacteria are found in marine and fresh water environments.

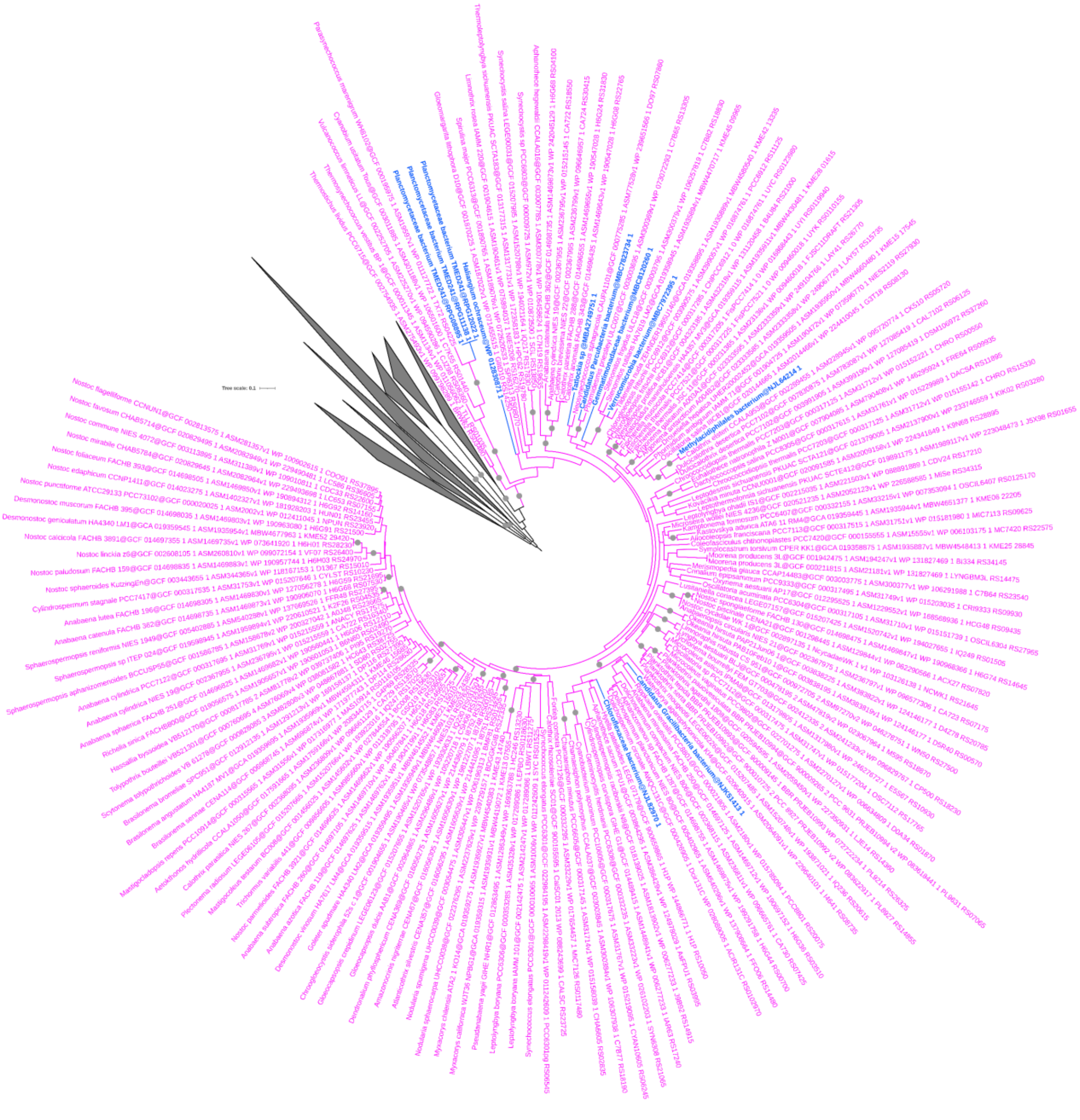
Tree scale: 0.1

Figure S10. Phylogeny of the 147 cyanobacterial GST1 sequences harboring the SRAS motif or related motifs.

The tree was inferred with *FastTree* [17] using the LG model. The scale bar indicates the average number of substitutions per site. Branches supported by SH-like values > 0.9 are indicated with gray circles. Sequences harboring the SRAS motif are indicated with filled triangles, while sequences harboring related motifs are designated by empty triangles. The taxonomy of the corresponding cyanobacterial proteomes is indicated: Gloeobacterales (brown), Synechococcales (orange), Pseudanabaenales (pink), Gloeomargaritales (dark blue), Thermostichales (black), Oscillatoriales (light green), Chroococcales (yellow), Pleurocapsales (purple), Chroococcidiopsidales (dark green), Nostocales (light blue), and unclassified (gray).

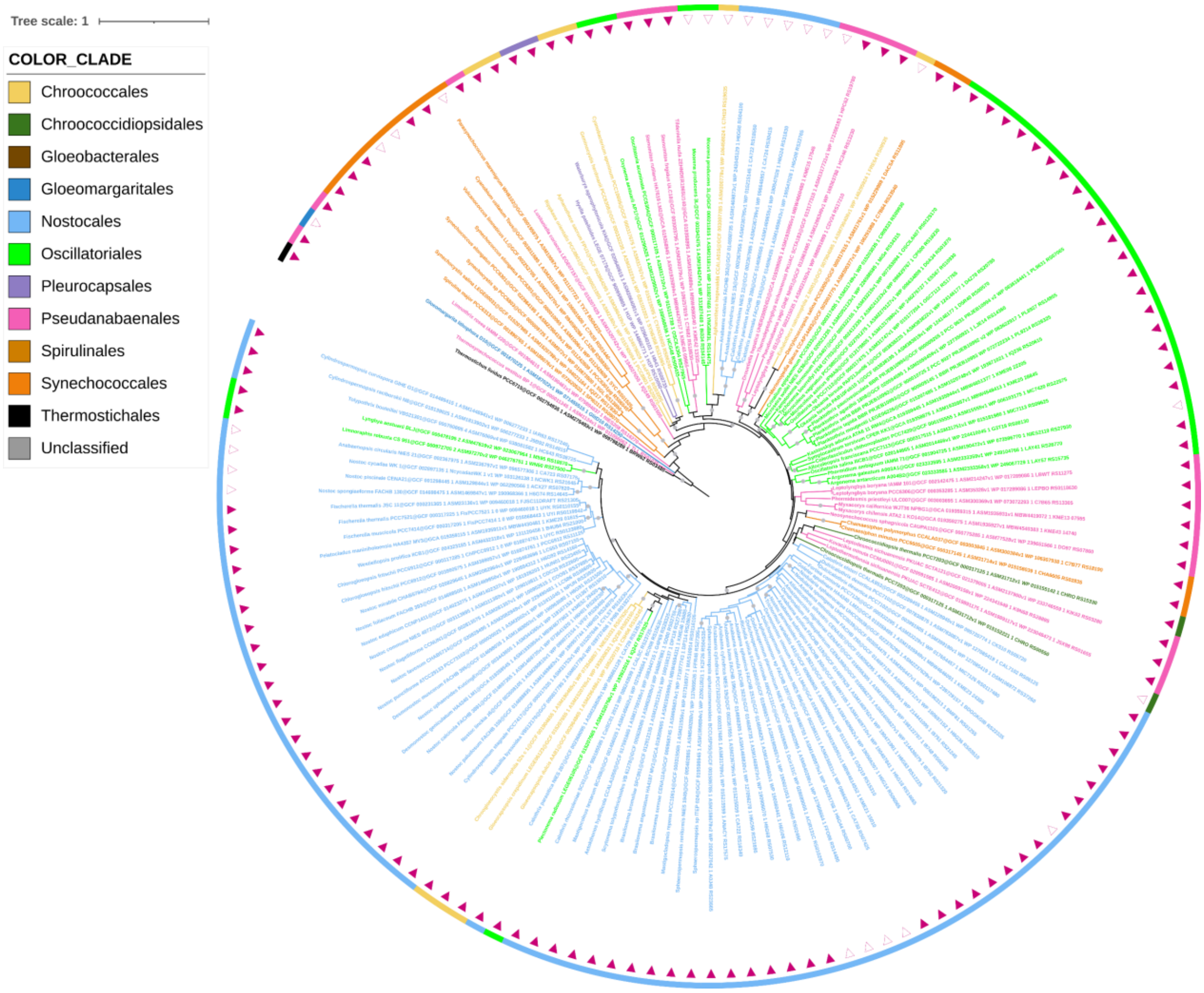
Tree scale: 1
COLOR_CLADE
Chroococcales
Chroococcidiopsidales
Gloeobacterales
Gloeomargaritales
Nostocales
Oscillatoriales
Pleurocapsales
Pseudanabaenales
Spirulinales
Synechococcales
Thermostichales
Unclassified

Figure S11. Phylogeny of the 222 proteomes of Cyanobacteria / Melainabacteria group considered in this study (high quality version of the tree provided in Figure 5).

The tree has been inferred with *IQ-TREE* [18] using the 52 rprots sequences present in more than 70% of the 222 proteomes (6,430 amino acid sites, LG+C20+F+R4 evolutionary model). The scale bar corresponds to the average number of substitutions per site. Gray circles correspond to ultrafast bootstrap values > 90% (1,000 replicates). The taxonomy of each proteome is indicated: Gloeobacterales (brown), Synechococcales (orange), Pseudanabaenales (pink), Gloeomargaritales (dark blue), Thermostichales (black), Oscillatoriales (light green), Chroococcales (yellow), Pleurocapsales (purple), Chroococcidiopsidales (dark green), Nostocales (light blue), and unclassified (gray). The 122 GSTC protein sequences harboring the SRAS motif are indicated with filled triangles, while the 25 GSTC sequences harboring variants of the SRAS motif are indicated with empty triangles. All the motifs are described in the Supplementary Table S8. The phylogeny of these 147 GSTC sequences is shown as Figure S10.

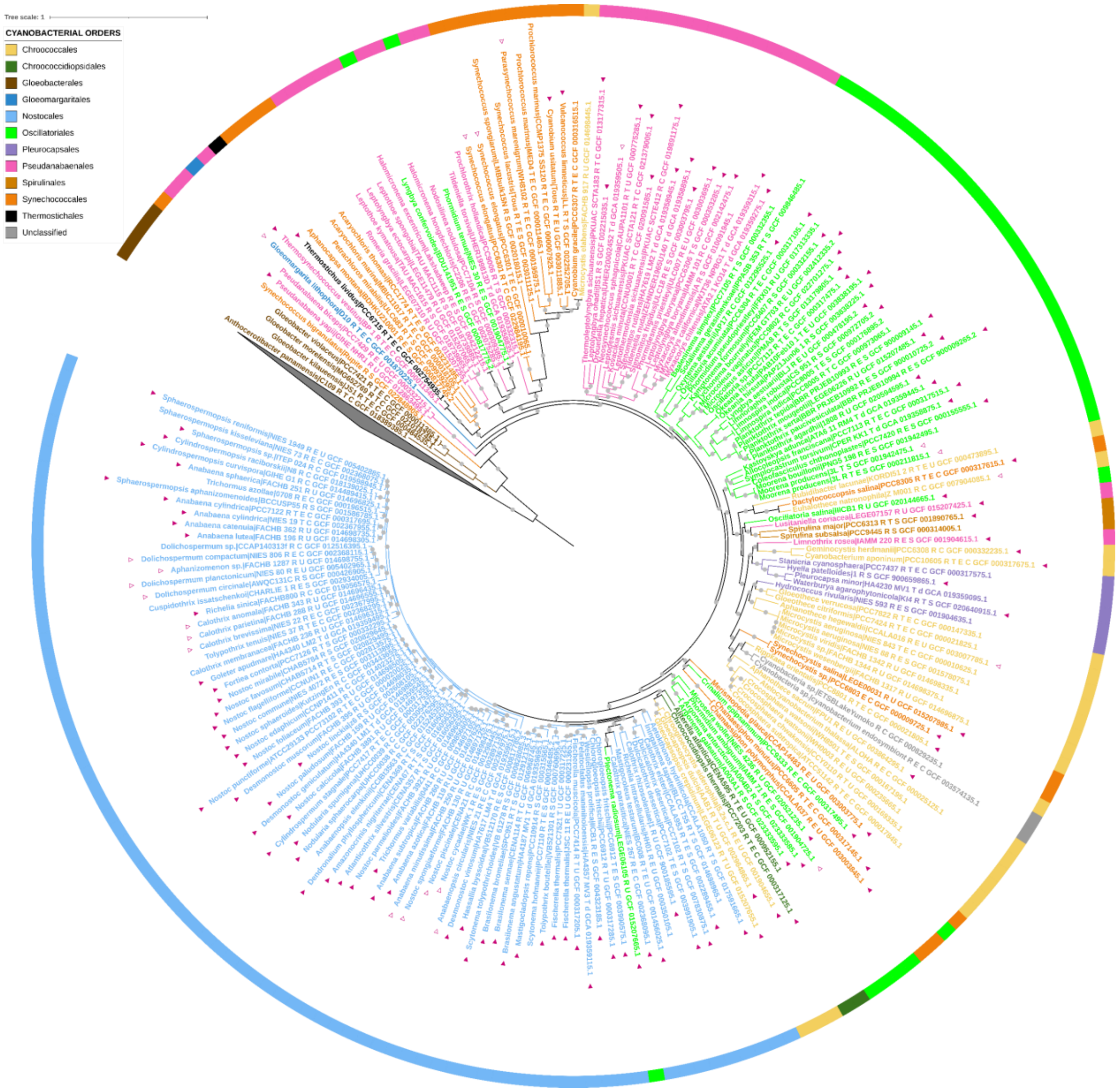
Tree scale: 1
CYANOBACTERIAL ORDERS
Chroococcales
Chroococcidiopsidales
Gloeobacterales
Gloeomargaritales
Nostocales
Oscillatoriales
Pleurocapsales
Pseudanabaenales
Spirulinales
Synechococcales
Thermostichales
Unclassified

Figure S12. Stereoview of the invariant amino acid residues in the GST Chi Class and WebLogos of aligned GSTCs from cyanobacteria.

A. The center of the monomer A is shown as a blue sphere. Only residues of the monomer A are represented for clarity. The invariant residues and S10 are shown as sticks and labelled. Residue numbering is based on SynGSTC1. The monomers A and B are shown in ribbon mode and colored green and blue, respectively. The secondary structures of the monomer A are labelled.

B. GSTC sequence logo was generated using WebLogo 3.7.11 (http://weblogo.threeplusone.com/create.cgi) [19] from 144 GSTC sequences displaying a SRAS or related motifs aligned with MAFFT v7.453 (https://mafft.cbrc.jp/alignment/server/) [20]. The overall height of the stack indicates the sequence conservation at that position, while the height of symbols within the stack indicates the relative frequency of each amino or nucleic acid at that position. The width of the stack is proportional to the fraction of valid symbols in that position. Error bars indicate an approximate Bayesian 95% confidence interval. Polar residues G,S,T,Y,C are colored in green, neutral (Q,N) in purple, basic (K,R,H) in blue, acidic (D,E) in red and hydrophobic (A,V,L,I,P,W,F,M) in black.

A.

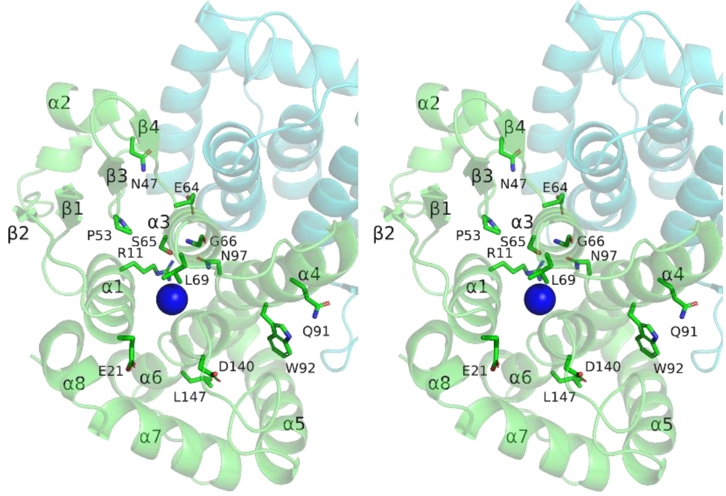

B.

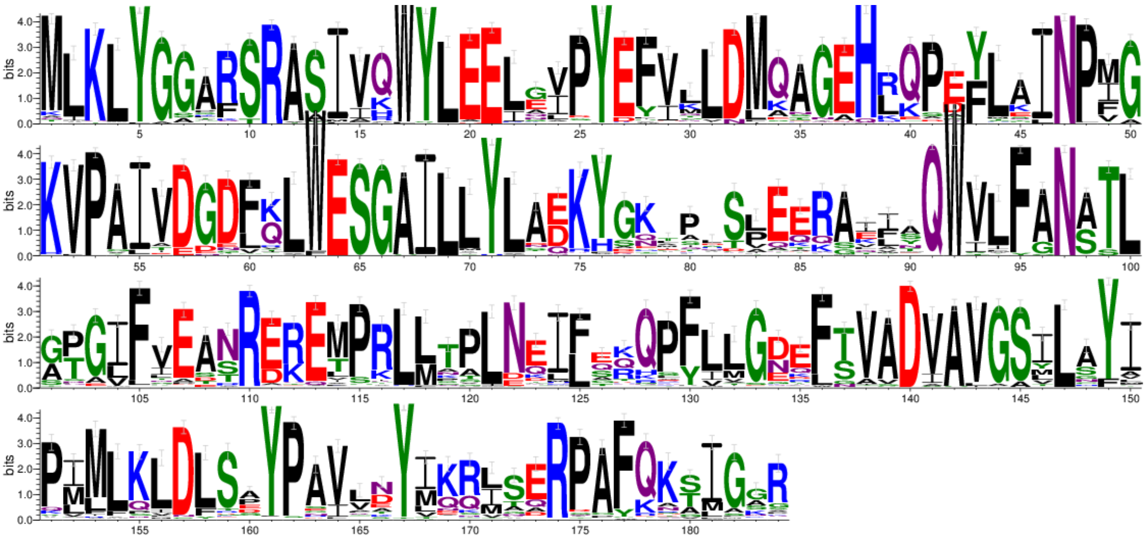

References.

1. Hansen, N.K.; Coppens, P. Testing aspherical atom refinements on small-molecule data sets. *Acta Crystallogr. Sect. A Found. Adv.* **1978**, *34*, 909–921. https://doi.org/10.1107/S0567739478001886.
2. Domagala, S.; Fournier, B.; Liebschner, D.; Guillot, B.; Jelsch, C. An improved experimental databank of transferable multipolar atom models—ELMAM2. Construction details and applications. *Acta Crystallogr. Sect. A Found. Adv.* **2012**, *68*, 337–351, https://doi.org/10.1107/S0108767312008197.
3. Guillot, B.; Enrique, E.; Huder, L.; Jelsch, C. MoProViewer: A tool to study proteins from a charge density science perspective. *Acta Crystallogr. Sect. A Found. Adv.* **2014**, *70*, C279. https://doi.org/10.1107/S2053273314097204.
4. Williams, C.J.; Headd, J.J., Moriarty, N.W., Prisant, M.G., Videau, L.L., Deis, L.N., Verma, V.; Keedy, D.A.; Hintze, B.J.; Chen, V.B.; et al. MolProbity: More and better reference data for improved all ‐ atom structure validation. *Protein Sci.* **2018**, *27*, 293–315. https://doi.org/10.1002/pro.3330.
5. DeLano, W.L. Pymol: An open-source molecular graphics tool. CCP4 Newsl. *Protein Crystallogr.* **2002**, *40*, 82–92.
6. Leduc, T.; Aubert, E.; Espinosa, E.; Jelsch, C.; Iordache, C.; Guillot, B. Polarization of Electron Density Databases of Transferable Multipolar Atoms. *J. Phys. Chem.* **2019**, *123*, 7156–7170. https://doi.org/10.1021/acs.jpca.9b05051.
7. Vuković, V.; Leduc, T.; Jelić-Matošević, Z.; Didierjean, C.; Favier, F.; Guillot, B.; Jelsch, C. A rush to explore protein–ligand electrostatic interaction energy with Charger. *Acta Crystallogr. Sect. D Biol. Crystallogr.* **2021**, *77*, 1292–1304. https://doi.org/10.1107/s2059798321008433.
8. Skopelitou, K.; Dhavala, P.; Papageorgiou, A.C.; Labrou, N.E. A glutathione transferase from *Agrobacterium tumefaciens* reveals a novel class of bacterial GST superfamily. *PLoS ONE* **2012**, *7*, e34263. https://doi.org/10.1371/journal.pone.0034263.
9. Roret, T., Thuillier, A., Favier, F., Gelhaye, E., Didierjean, C., Morel-Rouhier, M. Evolutionary divergence of Ure2pA glutathione transferases in wood degrading fungi. *Fungal Genet. Biol.* **2015**, *83*, 103–112. https://doi.org/10.1016/j.fgb.2015.09.002.
10. Sylvestre-Gonon, E.; Law, S.R.; Schwartz, M.; Robe, K.; Keech, O.; Didierjean, C.; Dubos, C.; Rouhier, N.; Hecker, A. Functional, Structural and Biochemical Features of Plant Serinyl-Glutathione Transferases. *Front. Plant Sci.* **2019**, *10*, 608. https://doi.org/10.3389/fpls.2019.00608.
11. Sheehan, D.; Meade, G.; Foley, V.M.; Dowd, C.A. Structure, function and evolution of glutathione transferases: Implications for classification of non-mammalian members of an ancient enzyme superfamily. *Biochem. J.* **2001**, *360*, 1–16. https://doi.org/10.1042/bj3600001.
12. Meux, E.; Prosper, P.; Masai, E.; Mulliert, G.; Dumarçay, S.; Morel, M.; Didierjean, C.; Gelhaye, E.; Favier, F. *Sphingobium* sp. SYK-6 LigG involved in lignin degradation is structurally and biochemically related to the glutathione transferase omega class. *FEBS Lett.* **2012**, *586*, 3944–3950. https://doi.org/10.1016/j.febslet.2012.09.036.
13. Aceto, A., Dragani, B., Melino, S., Allocati, N., Masulli, M., Ilio, C.D., Petruzzelli, R. Identification of an N-capping box that affects the α6-helix propensity in glutathione S-transferase superfamily proteins: A role for an invariant aspartic residue. *Biochem. J.* **1997**, *322*, 229–234. https://doi.org/10.1042/bj3220229.
14. Dirr, H.; Reinemer, P.; Huber, R. X-ray crystal structures of cytosolic glutathione Stransferases: Implications for protein architecture, substrate recognition and catalytic function. *Eur. J. Biochem.* **1994**, *220*, 645–661. https://doi.org/10.1111/j.1432-1033.1994.tb18666.x.
15. Emsley, P.; Lohkamp, B.; Scott, W.G.; Cowtan, K. Features and development of Coot. *Acta Crystallogr. Sect. D Biol. Crystallogr.* **2010**, 66, 486–501. https://doi.org/10.1107/s0907444910007493.
16. Smart, O.S.; Womack, T.O.; Flensburg, C.; Keller, P.; Paciorek, W.; Sharff, A.; Vonrhein, C.; Bricogne, G. Exploiting structure similarity in refinement: Automated NCS and target-structure restraints in BUSTER. *Acta Crystallogr. Sect. D Biol. Crystallogr.* **2012**, *68*, 368–380. https://doi.org/10.1107/S0907444911056058.
17. Price, M.N.; Dehal, P.S.; Arkin, A.P. FastTree 2--approximately maximum-likelihood trees for large alignments. *PLoS ONE* **2010**, *5*, e9490. https://doi.org/10.1371/journal.pone.0009490.
18. Minh, B.Q.; Schmidt, H.A.; Chernomor, O.; Schrempf, D.; Woodhams, M.D.; von Haeseler, A.; Lanfear, R. IQ-TREE 2: New Models and Efficient Methods for Phylogenetic Inference in the Genomic Era. *Mol. Biol. Evol.* **2020**, *37*, 1530–1534. https://doi.org/10.1093/molbev/msaa015.
19. Crooks, G.E.; Hon, G.; Chandonia, J.M.; Brenner, S.E. WebLogo: A sequence logo generator, *Genome Res.* **2004**, 14:1188-1190. http://doi.org/10.1101/gr.849004.
20. Katoh, K.; Standley, D.M. MAFFT multiple sequence alignment software version 7: Improvements in performance and usability. *Mol. Biol. Evol.* **2013**, 30, 772–780. https://doi.org/10.1093/molbev/mst010.
